# Critical Role of Molecular Packing in Lo Phase Membrane Solubilization

**DOI:** 10.3390/membranes13070652

**Published:** 2023-07-07

**Authors:** Nicolas Puff

**Affiliations:** 1 Faculté des Sciences et Ingénierie, Sorbonne Université, UFR 925 Physics, F-75005 Paris, France; nicolas.puff@sorbonne-universite.fr or nicolas.puff@univ-paris-diderot.fr; 2 Laboratoire Matière et Systèmes Complexes (MSC), UMR 7057 CNRS, Université Paris Cité, F-75013 Paris, France

**Keywords:** membrane solubilization, Triton X-100, liquid-ordered phase, Laurdan, LUVs

## Abstract

Membrane solubilization induced by Triton X-100 (TX-100) was investigated. Different membrane compositions and phase states were studied along the detergent titration. Expected solubilization profiles were obtained but new information is provided. The fluorescence of nitrobenzoxadiazole (NBD)-labeled lipids indicates that the liquid-ordered (Lo)/liquid-disordered (Ld) phase coexistence is barely unaffected at sub-solubilizing detergent concentrations and highlights the vesicle-to-micelle transition. Moreover, the location of the NBD group in the bilayer emphasizes a detergent–membrane interaction in the case of the insoluble Lo phase membrane. It has also been shown that the molecular packing of the membrane loosens in the presence of TX-100, regardless of the solubilization profile. Motivated by studies on GPMVs, the solubilization of less ordered Lo phase membranes was considered in order to improve the effect of molecular packing on the extent of solubilization. Membranes composed of SM and Chol in an equimolar ratio doped with different amounts of PC were studied. The more ordered the Lo phase membrane is in the absence of detergent, the less likely it is to be solubilized. Furthermore, and in contrast to what is observed for membranes exhibiting an Lo/Ld phase coexistence, a very small decrease in the molecular packing of the Lo phase membrane radically modifies the extent of solubilization. These results have implications for the reliability of TX-100 insolubility as a method to detect ordered domains.

## 1. Introduction

Cellular plasma membranes are mosaics of different types of domains with different sizes, compositions, dynamics, and functions [1,2,3,4]. Among the different types of membrane domains, several are based on lipid interactions, the best documented being so-called lipid rafts [5,6]. Rafts are thought to be rich in sphingolipids, cholesterol (Chol), and specific proteins and to be in a phase state distinct from the surrounding membrane [7,8]. These domains are considered to be highly dynamic nanoscale assemblies [9,10,11,12] and are postulated to be involved in many biological functions such as cellular activation, membrane trafficking, and signal transduction.

The raft hypothesis and its physicochemical nature are closely related to the existence of detergent-resistant membranes (DRMs). The insolubility of membrane components in detergents is assumed to be related to their localization in raft domains [13,14]. However, due to the conditions used for their isolation, DRMs are unlikely to have the same composition as plasma membrane lipid rafts at physiological temperature. The composition of DRMs is indeed detergent and protocol dependent and cannot be directly associated with lipid rafts [15,16]. Despite these shortcomings, DRMs remain an important biochemical tool for assessing the potential affinity of some molecules for rafts [17] and have undoubtedly contributed to the understanding of the lateral organization of plasma membranes.

In parallel to studies of DRMs isolated from cells, artificial model membranes have been developed and used to elucidate the fundamental properties of lateral heterogeneities in membranes. Indeed, it has been proposed that plasma membrane lipids contribute to the lateral organization of membranes via the same thermodynamic forces that drive the liquid-ordered (Lo)–liquid-disordered (Ld) phase separation in model membranes composed only of lipids [18,19]. Many studies have focused on the mechanisms of solubilization of lipid membranes induced by detergents, and in particular by the one most commonly used in cellular studies, Triton X-100 (TX-100) [15,20,21,22]. It has been shown that the extent of solubilization is strongly dependent on the lipid composition, the temperature, and thus the membrane phase. According to the classical three-stage model [23], membranes in gel or Ld phases could be completely solubilized at strong enough detergent to lipid ratios [24,25]. On the other hand, Lo phase membranes composed of sphingomyelin and cholesterol were found to be highly resistant to solubilization [26,27]. When liquid phases coexist within the membrane of a giant unilamellar vesicle (GUV), it has been shown that the addition of TX-100 induces solubilization of the single Ld phase, leaving an insoluble daughter vesicle in an apparently unaffected Lo phase [28,29]. This partial membrane solubilization has been extensively studied in a recent work where the extent of solubilization was quantified over a wide compositional range of “raft-like” lipid mixtures [22]. Again, Lo phase domains in GUVs remain insoluble and the extent of solubilization decreases significantly as the fractions of SM and Chol in the mixture increase. This preferential solubilization of the Ld phase over the Lo one is thought to be due to the tight packing of the Lo phase, which hinders the insertion of the detergent and its subsequent solubilization. There is also evidence that the detergent itself causes an important lateral rearrangement within the membrane in sub-solubilizing amounts, increasing the phase diagram region of microscopic Lo/Ld phase coexistence. It was originally proposed that TX-100 induces domain formation by reducing the miscibility of sphingomyelin (SM) with molecules concentrated within the Ld phase (TX-100 and unsaturated lipids) [30]. Recent works would rather suggest that these domains are formed by the coalescence of pre-existing submicroscopic domains [22,31]. TX-100 insertion increases the interfacial energy at the domain boundaries, leading to domain coalescence, boundary length reduction, and thus to a decrease in interfacial free energy [32].

Studies on giant plasma membrane vesicles (GPMVs)—in which liquid–liquid phase coexistence was also observed [33]—have also shown a correlation between the presence of a separate ordered phase and detergent resistance [34,35]. GPMVs capture much of the compositional protein and lipid complexity of intact cell plasma membranes and provide an alternative to vesicles constructed from synthetic or purified lipids. Interestingly, it has been shown that the physicochemical properties of ordered and disordered domains can differ significantly between model systems and GPMVs. The ordered phase in GPMVs is less ordered than the Lo domains in model membranes, while the disordered phase of cell-derived membranes is more packed than the Ld phase of model membranes [36,37]. This leads to very different ΔGP values between the two systems ($\Delta GP=GP_{ordered}-GP_{disordered}$), raising the question of the relevance in membrane solubilization studies of some of the model systems used, especially those concerning the Lo phase solubilization.

In the present work, membrane solubilization induced by TX-100 was investigated. Different membrane compositions and phase states were studied along the detergent titration. The expected solubilization profiles were obtained [15,22,25,27] but the fluorescence spectroscopy experiments provide new information. Another goal of this work, motivated by studies conducted on GPMVs, was to consider the solubilization of less ordered Lo phase membranes in order to improve the effect of molecular packing on the extent of solubilization. The more ordered the Lo phase membrane in the absence of detergent, the less likely it is to be solubilized. Furthermore, and in contrast to what is observed for membranes exhibiting an Lo/Ld phase coexistence, a very small decrease in the molecular packing of the Lo phase membrane radically modifies the extent of solubilization. These results have implications for the reliability of TX-100 insolubility as a method to detect ordered domains.

## 2. Materials and Methods

### 2.1. Chemicals

Egg yolk L-α-phosphatidylcholine (PC), egg yolk sphingomyelin (SM), cholesterol (Chol), and the lipophilic membrane probe C${}_{12}$NBD-PC (1-acyl-2-[12-[(7-nitrobenz-2-oxa-1,3-diazol-4-yl)amino]dodecanoyl]-sn-glycero-3-phosphocholine) were from Avanti Polar Lipids and used without further purification. The fluorescent probe 6-dodecanoyl-2- dimethylaminonaphthalene (Laurdan) was from Molecular Probes Inc. (Eugene, OR, USA). All others chemicals were of highest purity grade.

### 2.2. Large Unilamellar Vesicle Preparation

LUVs were prepared using the extrusion method [38]. Samples were prepared by dissolving and mixing the indicated lipids in chloroform/methanol (9.4:0.6 *v*/*v*) to obtain the desired compositions. Thereafter, the solvent was removed under a stream of oxygen-free dry nitrogen (20 min). The residues were subsequently maintained under vacuum for 2 h and then HEPES buffer, pH 7.4 (HEPES 5 mM, EDTA 0.1 mM), was added at room temperature to yield a lipid concentration of 2 mM. The samples were heated at 60 °C for 30 min, vortexed for 2 min, left for a few minutes in a sonication bath, vortexed again for 1 min to ensure more uniform vesicle dispersion, and incubated again at 60 °C for 15 min. Lipids are assumed to be stable under these conditions [39,40]. The use of temperatures above the main phase transition and phase separation of lipids aimed to obtain a homogeneous lipid mixture for the subsequent LUV preparation. The multilamellar vesicles obtained at this stage were then extruded with a LiposoFast small-volume extruder equipped with polycarbonate filters (Avestin, Ottawa, ON, Canada) as follows: 12 extrusions through 800 nm, followed by 21 extrusions through 100 nm filters. The fluorescent probes Laurdan and C${}_{12}$NBD-PC were, respectively, mixed with the lipids in the initial organic solution at probe/lipid ratios of 1:200 and 2:100. The different LUV samples, those with Laurdan and the others with NBD-labeled PC, were kept at 4 °C and used the following day.

### 2.3. Light Scattering Measurements

To determine the TX-100 concentrations that induce membrane solubilization for the various systems studied here, 90° static light scattering (SLS) was performed on LUVs. Light scattering is a sensitive indicator of changes in the size of the structure, as the amount of light scattered depends on the sixth power of the radius of the suspended particles. Measurements were performed in a Cary Eclipse spectrofluorometer (Agilent Technologies Inc., Santa Clara, CA, USA)). LUVs were incubated with TX-100 at different detergent/lipid ratios for 30 min at 20 °C, and the final lipid concentration was 0.1 mM. The different suspensions of liposomes were placed in a quartz cuvette with a 1 cm light path. After the incubation time, the 90° light scattering intensity at λ= 600 nm was measured directly for each sample at 20 °C. Three measurements per TX-100 concentration were performed to determine the intrinsic accuracy of the method. The values obtained for each detergent-to-lipid molar ratio were normalized with respect to the light scattering of the suspension of LUVs without TX-100. For each system studied, the normalized intensity value obtained at the highest TX-100 concentration studied (1.712 mM) was used to estimate the so-called “residual insoluble fraction” (RIF).

Dynamic light scattering (DLS) measurements were also performed along the titration of LUVs with TX-100 under exactly the same experimental conditions as for the SLS experiments. DLS is widely used to determine the size distribution of nanoscale particles suspended in an aqueous solvent. In this technique, the detected scattered light is fed to a signal processing correlator which provides the intensity autocorrelation function from which the diffusion constant and then the average size are obtained. The polydispersity index (PdI)—a dimensionless measure of the broadness of the DLS size distribution—is also provided. DLS experiments were performed using the Zetasizer Nano ZS (Malvern Panalytical Ltd., Grovewood, UK). All DLS data were collected using 178° backward scattering and averaged over four experimental runs, each of which was summed up over twelve time correlograms fitted by the Zetasizer software.

### 2.4. Fluorescence Measurements

Steady-state fluorescence measurements were performed with a Cary Eclipse spectrofluorimeter (Agilent Technologies Inc., Santa Clara, CA, USA) equipped with a thermostated cuvette holder (±0.1 °C). Excitation and emission slits were set to 5 nm.

#### 2.4.1. Lipid Aggregate Structure at the Molecular Level

To follow the changes in vesicle structure and micellization at the molecular level, fluorescence measurements were performed, respectively, with two fluorescent probes: an NBD-labeled lipid and Laurdan. Prior to measurements, LUVs were incubated with TX-100 at different detergent/lipid ratios for 30 min at 20 °C. The final lipid concentration was 0.1 mM. Fluorescence emission spectra and intensities were all recorded at 20 °C.

During the membrane titration with TX-100, the variation in the C${}_{12}$NBD-PC fluorescence intensity maximum was followed. It is known that NBD fluorescence intensity is modulated by two mechanisms: (i) the effect of the environment on NBD fluorescence, i.e., the composition and structure of the lipid aggregate [41,42], and (ii) concentration-dependent NBD self-quenching, i.e., by the concentration of the NBD fluorophore in the lipid aggregate [43]. It is therefore a useful tool for studying the membrane structure and heterogeneity as well as a likely relevant indicator of the vesicle-to-micelle transition. C${}_{12}$NBD-PC fluorescence was excited at 470 nm, and the emitted fluorescence maximum was measured at 538 nm. The reproducibility of specific fluorescence intensity measurements between carefully prepared samples was ±8%. This error increased to ±12% for membranes with Lo–Ld phase coexistence, possibly because small variations in cholesterol content can cause significant differences in the overall fluorescence intensity.

Laurdan is a fluorescent probe that is sensitive to the polarity of its environment. Variations in the water content of the probe environment cause shifts in the Laurdan emission spectrum, which are quantified by calculating the generalized polarization (GP). In addition to its well-known ability to account for membrane order, Laurdan has been shown to be sensitive also to the vesicle-to micelle transition [44]. Consequently, GP was measured along the LUV titration with TX-100. The GP is defined as $GP=I_{440}-I_{490}/I_{440}+I_{490}$, where $I_{440}$ and $I_{490}$ are the emission intensities at 440 and 490 nm, respectively. GP measurements were made by simply registering the two emission intensities mentioned above (two sets of five measurements averaged).

#### 2.4.2. Membrane Phase State

In order to reveal the membrane phase state at the titration temperature (20 °C), a temperature scan of cooling was performed with several GP measurements from 60 to 15 °C. It was verified that (i) there was no hysteresis when the temperature scan was performed heating the sample from 15 to 60 °C, and (ii) the probe emission intensity was stable with time after 10 min of equilibration, assuming that the system had reached a steady state. As previously described [45], LUVs that remain in a single phase over the entire temperature range yield GP vs. T curves with a single concavity. On the other hand, LUVs that undergo a phase transition yield a change in concavity with an inflection point corresponding to the miscibility transition temperature ($T_{misc}$). These temperatures were determined by fitting the GP versus temperature curve to the Boltzmann sigmoid function given by Equation (1) [46], where $GP_{1}$ and $GP_{2}$ are the upper and lower values of GP, $T_{misc}$ is the inflection point, and ΔT is the specific slope at $T=T_{misc}$ [45].

(1) $$GP=GP_{2}+\frac{GP_{1}-GP_{2}}{1+\exp\left(\frac{T-T_{misc}}{\Delta T}\right)}$$

## 3. Results

**Figure A1:**
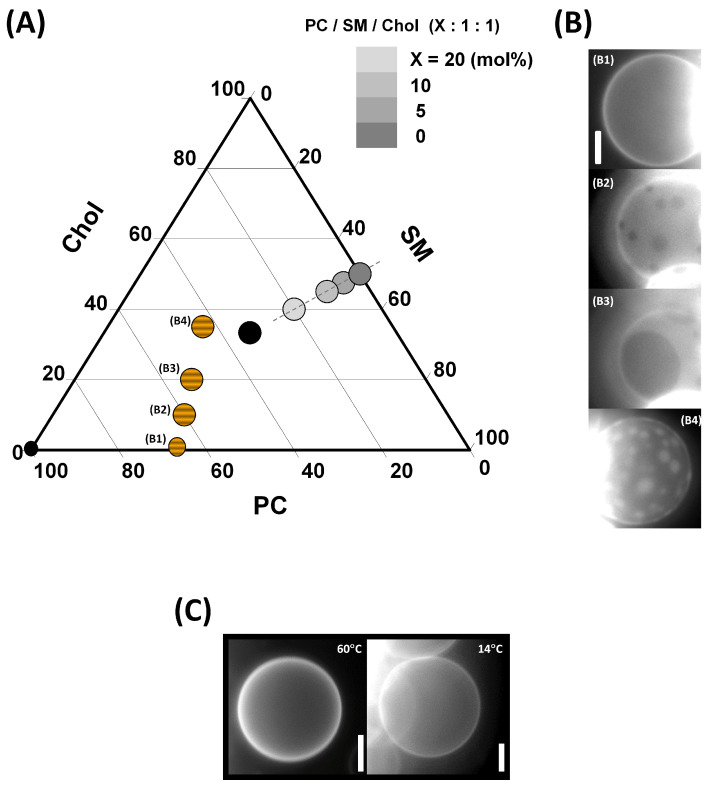
(**A**) Compositional phase diagram of the PC/SM/Chol mixtures. The studied compositions are indicated by symbols (striped filled circles for lipid compositions whose membrane was specifically imaged by fluorescence microscopy, gray filled circles of different shades for PC-doped Lo phases, and black filled circles for the other lipid compositions). (**B**) Images of GUVs taken at 20 °C. B1–B4: GUV formed from PC/SM, 2:1, containing 4 mol% C${}_{12}$NBD-PC and, B1, 0 mol%; B2, 10 mol%; B3, 20 mol%; B4, 35 mol% cholesterol. Adapted with permission from Coste et al. [42], Biochim. Biophys. Acta Biomembranes; published by Elsevier, 2006. (**C**) Fluorescence microscopic images of GUVs containing 0.8 mol% Rhod-PE with a SM/Chol 50:50 (mol/mol) composition at two temperatures. Adapted with permission from Puff et al. [45], Langmuir; published by the ACS, 2020. Scale bars represent 20 µm.

The effects of TX-100 detergent on lipid vesicles were investigated as a function of the membrane composition. Pure, binary, and ternary lipid compositions were studied (see the compositional phase diagram in Figure A1 in Appendix A) to assess the role of the membrane phase and order on membrane solubilization by TX-100. Due to its mechanism of action (fast transbilayer movement), TX-100 is considered as a fast detergent [47,48]. For this reason, LUVs were incubated with TX-100 for 30 min at 20 °C before performing the corresponding measurements.

### 3.1. Vesicle-to-Micelle Transition

**Figure 1:**
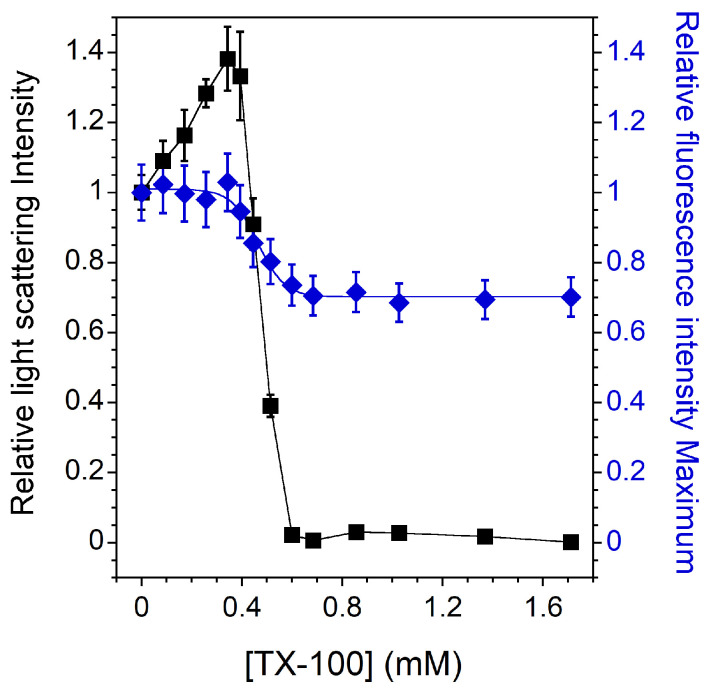
Relative 90° light scattering intensity (*▪*) and C${}_{12}$NBD-PC fluorescence intensity maximum (⧫) as a function of the TX-100 concentration for 2 mol% C${}_{12}$NBD-PC containing PC LUVs in the Ld phase. All measurements were performed at a lipid concentration of 0.1 mM and were recorded at 20 °C. For the light scattering experiments, error bars are shown for values that have been measured three times. The reproducibility of the fluorescence intensity measurements between carefully prepared samples was ±8%.

Figure 1 shows the relative light scattering intensity and the C${}_{12}$NBD-PC fluorescence intensity maximum of pure PC vesicles as a function of the detergent concentration. The intensities shown in Figure 1 are relative to the initial values of light scattering and C${}_{12}$NBD-PC fluorescence intensities obtained in the absence of TX-100. For PC LUVs in the Ld phase at 20 °C ($T_{m}$ around −5 °C [49]), the three stages of solubilization are very well defined. First, during the incorporation of TX-100 into the vesicles, an increase in the relative light scattering intensity is observed, which could indicate an initial swelling of the liposomes and/or some fusion events. At the same time, there is no significant change in the relative C${}_{12}$NBD-PC fluorescence intensity maximum, indicating that the environment of the probe embedded in the bilayer does not change significantly [42]. Then, the light scattering intensity of the sample steadily decreases as lipids and detergents are gradually transferred from LUVs to mixed micelles. During this process, the emitted fluorescence maximum also decreases. Indeed, the vesicle-to-micelle transition results in greater penetration of water molecules into more curved mixed micelles, thereby increasing the polarity in the NBD environment with a consequent decrease in NBD fluorescence intensity [50]. Finally, the relative light scattering reaches almost zero, indicating that the solubilization is complete and that only mixed micelles are present. At this stage, the C${}_{12}$NBD-PC relative fluorescence intensity maximum remains constant, indicating that the environment of the probe embedded in the mixed micelles is no longer changing.

### 3.2. Solubilization of Membranes Exhibiting Lo/Ld Phase Coexistence

**Figure A2:**
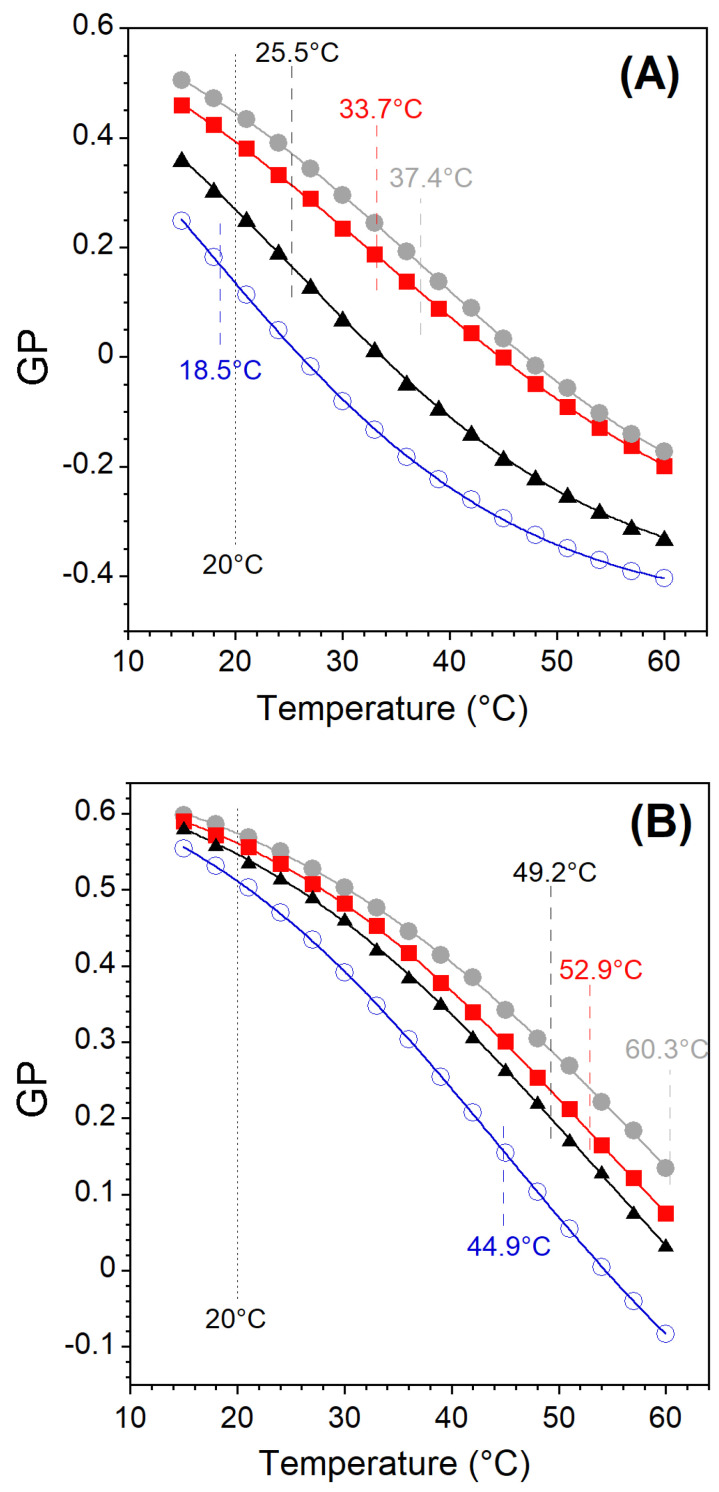
Temperature dependence of the Laurdan GP of LUVs with different lipid compositions (Table 1 and Table 2). Miscibility transition temperatures are given in the figures, as well as the titration temperature. (**A**) Lo-Ld 1 (open blue circles), Lo-Ld 2 (black triangles), Lo-Ld 3 (red filled squares), Lo-Ld 4 (gray filled circles). (**B**) Lo 1 (gray filled circles), dLo 1 (red filled squares), dLo 2 (black triangles), dLo 3 (open blue circles).

The effect of TX-100 on LUVs composed of four different PC/SM/Chol mixtures (Table 1) is now investigated. GUV fluorescence microscopy experiments show that Lo-Ld samples 1 to 3 exhibit Lo/Ld phase coexistence at 20 °C (Figure A1). LUV fluorescence spectroscopy experiments confirm these results and show that Lo-Ld sample 4 also exhibits a liquid–liquid phase coexistence at 20 °C (Figure A2). In fact, the temperature at which the solubilization experiments are performed is lower than the miscibility transition temperatures $T_{misc}$ determined here (Table 1). Fluorescence microscopy images of GUVs also show that increasing the cholesterol content at a constant PC/SM molar ratio induces a higher Lo surface fraction (Figure A1 and Table 1, $\Phi_{L_{o}}$). As expected, this increasing surface fraction in the Lo phase corresponds to a higher membrane order (Table 1, $GP_{ini}$ for Lo-Ld samples 1–3). Among the four raft-like lipid mixtures studied, the particular 1:1:1 PC/SM/Chol mixture has the highest membrane order (Table 1, $GP_{ini}$ for Lo-Ld sample 4).

**Figure 2:**
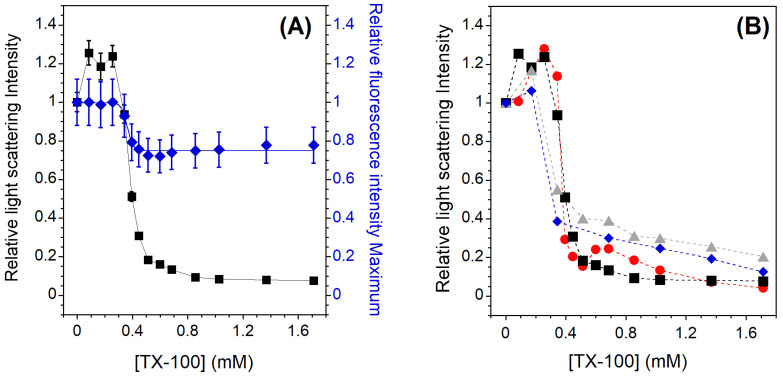
(**A**) Relative 90° light scattering intensity (*▪*) and C${}_{12}$NBD-PC fluorescence intensity maximum (⧫) as a function of the TX-100 concentration for 2 mol% C${}_{12}$NBD-PC containing LUVs exhibiting phase coexistence (Lo-Ld sample 2). For the light scattering experiments, error bars are shown for values that have been measured three times. The reproducibility of the fluorescence intensity measurements between carefully prepared samples was ±12%. (**B**) Relative 90° light scattering intensity as a function of the TX-100 concentration for Lo-Ld 1 (•), Lo-Ld 2 (*▪*), Lo-Ld 3 (⧫), and Lo-Ld 4 (▴) LUVs. Error bars are not shown here for clarity. All measurements were performed at a lipid concentration of 0.1 mM and were recorded at 20 °C.

**Figure A3:**
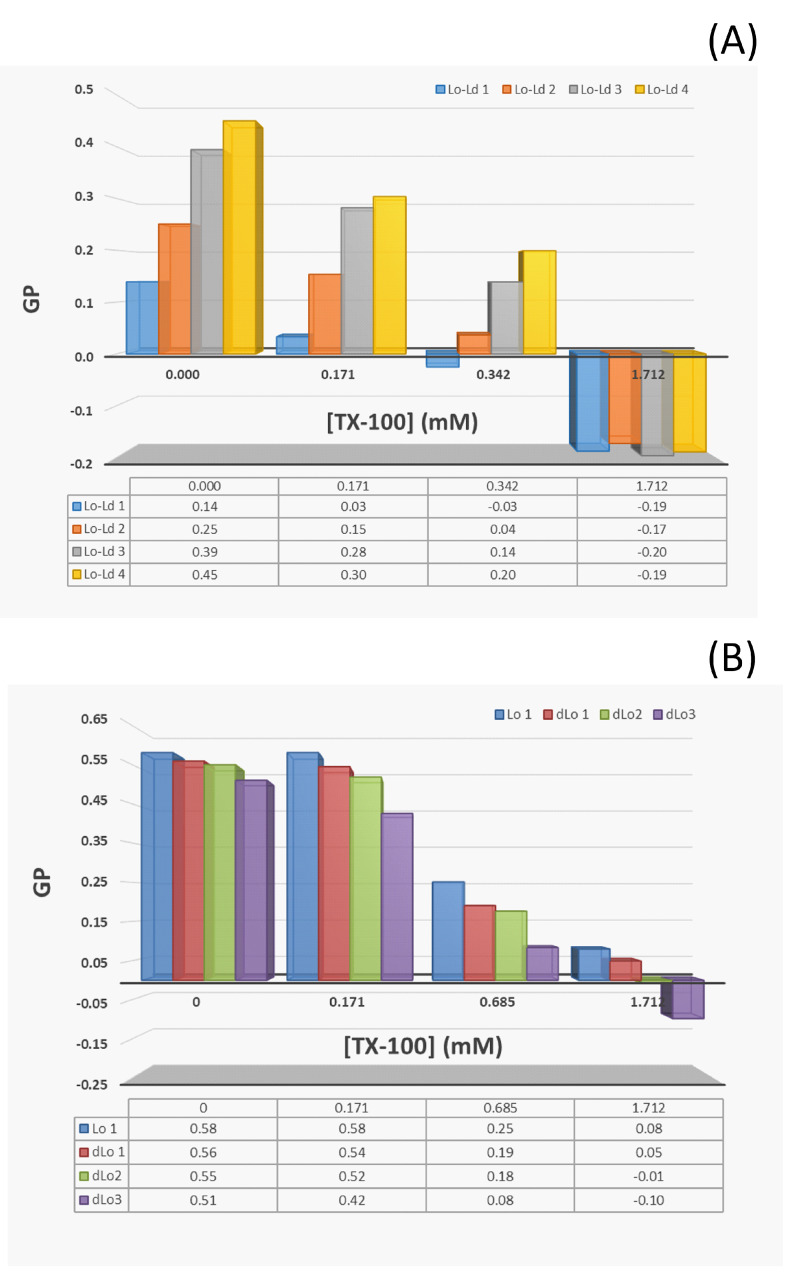
Laurdan GP values of different membranes as a function of the TX-100 concentration: (**A**) Lo-Ld lipid mixtures (Table 1), (**B**) Lo and dLo lipid mixtures (Table 2). The color codes are indicated in the figures.

For all the Lo-Ld samples studied, the solubilization profile induced by the detergent shows a behavior similar to that observed in the case of the membrane in the Ld phase. An example is shown in Figure 2A for the Lo-Ld lipid mixture 2 (Table 1). The three stages described previously are still present but the onset of solubilization is slightly shifted to a lower TX-100 concentration (0.3 instead of 0.4 mM), and incomplete solubilization is observed since the relative light scattering intensity does not reach zero. It is worth noting that the relative C${}_{12}$NBD-PC fluorescence intensity maximum remains constant at sub-solubilizing concentrations of TX-100, indicating that phase coexistence is barely unaffected by detergent incorporation (Figure 2A). Indeed, the Lo/Ld phase coexistence can be monitored by the concentration-dependent self-quenching of C${}_{12}$NBD-PC, which is known to be largely excluded from the Lo phase [42]. The constant intensity therefore indicates that the relative Lo and Ld membrane fractions remain approximately the same, although on average the membrane packing loosens during detergent incorporation (GP initially at 0.25 falls to 0.15 at 0.171 mM TX-100, Figure A3). After the onset of solubilization, the emitted fluorescence maximum behaves as in the case of PC vesicles, as it decreases during the vesicle solubilization and then reaches a constant value. In contrast to PC vesicles, the light scattering intensity here does not reach a value equal to zero (Figure 2A), indicating that even at high TX-100 concentrations, residual insoluble membrane fragments larger than micelles could remain in suspension.

Figure 2B shows the relative light scattering intensities for all studied Lo-Ld lipid mixtures. For the Lo-Ld sample 2, and in agreement with the work of Caritá et al. [22], the membrane solubilization process is not complete. A partial insolubility of the membrane is observed, which is more pronounced when the cholesterol content is high (Table 1, RIF). This is probably due to the to the tight packing of the Lo phase, which hinders the insertion of the detergent and its subsequent solubilization [26,27].

The evolution of the membrane molecular packing induced by the addition of TX-100 is followed by the measurements of the Laurdan GP. At the sub-solubilizing TX-100 concentration of 0.171 mM, the measured GP value is lower than that without detergent (Figure A3). This decrease is due to the loosening of the membrane packing caused by the detergent incorporation. During the solubilization process (0.342 mM TX-100), the GP value still decreases. This value takes into account the environment of Laurdan, which is present here in both in vesicles and in insoluble fragments. At the highest TX-100 content (1.712 mM), there are no more LUVs and the GP value is even lower (Figure A3), which is consistent with the more polar environment of Laurdan due to the greater curvature of the membrane-insoluble fragments (e.g., worm-like micelles) relative to the vesicles [44]. At this TX-100 concentration, it can be seen that the molecular packing of the aggregates is more or less the same regardless of the lipid mixture (Table 1, $GP_{fin}$).

### 3.3. Lo and PC-Doped Lo Phase Membrane Solubilization

**Figure 3:**
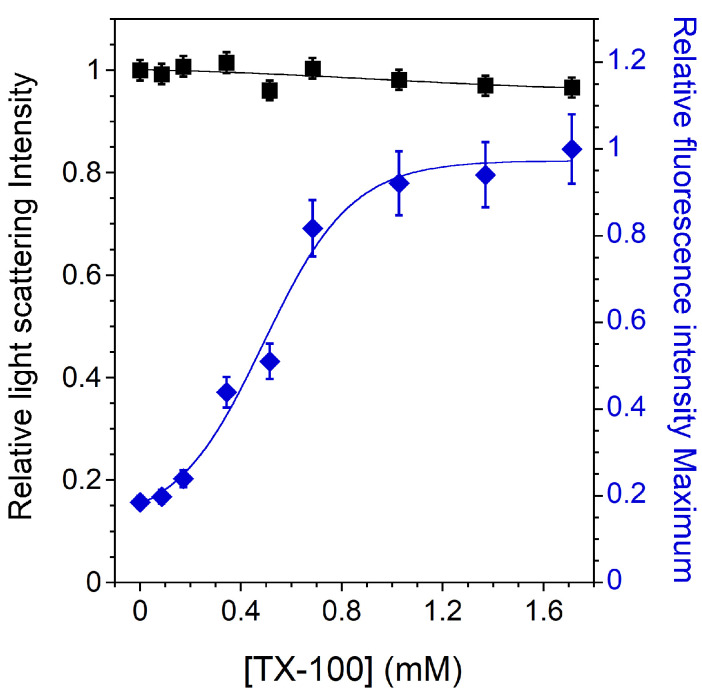
Relative 90° light scattering intensity (*▪*) and C${}_{12}$NBD-PC fluorescence intensity maximum (⧫) as a function of the TX-100 concentration for 2 mol% C${}_{12}$NBD-PC containing LUVs (Lo lipid mixture 1). All measurements were performed at a lipid concentration of 0.1 mM and were recorded at 20 °C. For the light scattering experiments, error bars are shown for values that have been measured three times. The reproducibility of the fluorescence intensity measurements between carefully prepared samples was ±8%.

Figure 3 shows the relative light scattering intensity and the C${}_{12}$NBD-PC fluorescence intensity maximum as a function of detergent concentration for an Lo phase bilayer composed of SM and Chol in an equimolar ratio (Table 2, Lo 1 mixture). SM/Chol 1:1 was chosen as a consistent Lo phase composition in the investigated temperature range [51,52]. GUV fluorescence microscopy and LUV fluorescence spectroscopy experiments confirm this result, as fluorescent GUVs remain homogeneous between 14 and 60 °C (Figure A1) and the measured $T_{misc}$ is higher than 20 °C (Figure A2 and Table 2, $T_{misc}$).

As observed in several studies for similar systems [22,27,53], there is no noticeable decrease in the light scattering signal, indicating that the membrane is almost completely insoluble (Figure 3). Increasing the incubation time does lead to a decrease in the light scattering intensity, indicating a very poor solubilization efficiency of TX-100 for lipids in the Lo phase [22]. The tight packing of the Lo phase is generally cited as an obstacle to detergent insertion and the subsequent solubilization of such a membrane.

However, the variation in the C${}_{12}$NBD-PC fluorescence signal with TX-100 content still indicates a detergent–membrane interaction. This has already been observed in a similar insoluble SM/Chol membrane, where TX-100 induced permeabilization and some fluorescent lipid extraction was observed [53]. Contrary to what was observed for previous mixtures, it can be seen in Figure 3 that the C${}_{12}$NBD-PC fluorescence intensity maximum increases as the amount of TX-100 increases, and does not follow the solubilization profile of the membrane. This increase indicates that the NBD group senses an increasingly apolar environment in the insoluble membrane. Indeed, the NBD group of the acyl chain labeled-PC, known to loop up to the membrane interface in fluid phases [41,54], is located in a more external position in an ordered phase membrane than in a disordered one, thus exposing the NBD group to more water [42,55]. This shallower location of the NBD group in the Lo phase membrane corresponds well to the low intensity value measured without TX-100. Then, as the membrane becomes increasingly less packed (Figure A3), the environment of the NBD group becomes progressively more apolar, consistent with its progressively deeper location. Despite the insolubility of the membrane, the gradual decrease in the GP in the presence of TX-100 again supports the idea of a detergent–lipid interaction.

The same type of experiments were performed for PC-doped Lo LUVs composed of three different PC/SM/Chol mixtures (Table 2, dLo samples 1–3). LUV fluorescence spectroscopy experiments with Laurdan showed that these membranes are also in the Lo phase at 20 °C regardless of the PC content (Figure A2 and Table 2, $T_{misc}$). Furthermore, it can be seen that the less packed the membrane is, the higher the C${}_{12}$NBD-PC fluorescence intensities are (Table 2, $GP_{ini}$ and $I_{ini}$), again demonstrating that membrane packing significantly affects the location of the NBD group.

**Figure 4:**
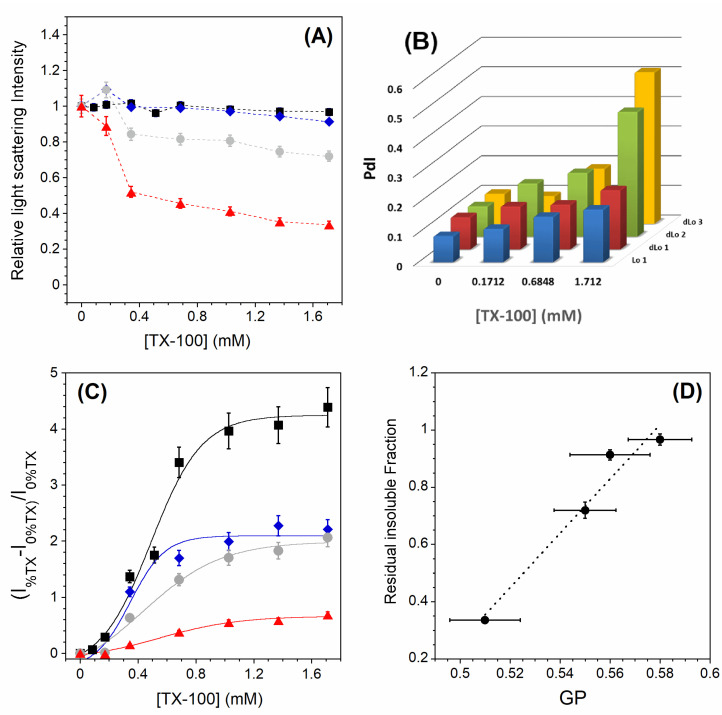
(**A**) Relative 90° light scattering intensity as a function of the TX-100 concentration for Lo 1 (*▪*), dLo 1 (⧫), dLo 2 (•), and dLo 3 (▴) LUVs. Error bars are shown for values that have been measured three times. (**B**) Polydispersity index PdI (a dimensionless measure of the broadness of the DLS size distribution) as a function of the TX-100 concentration. (**C**) C${}_{12}$NBD-PC relative normalized intensity for 2 mol % C${}_{12}$NBD-PC in LUVs of different compositions. Lo 1 (*▪*), dLo 1 (⧫), dLo 2 (•), and dLo 3 (▴). C${}_{12}$NBD-PC fluorescence was excited at 470 nm and the emitted fluorescence maximum was measured at 538 nm. The reproducibility of the fluorescence intensity measurements between carefully prepared samples was ±8%. (**D**) Residual insoluble fractions vs. GP values measured without TX-100. The RIF error bars are shown for values that have been measured three times and the GP error bars are shown for values measured in two sets of five measurements. All measurements were performed at a lipid concentration of 0.1 mM and were recorded at 20 °C.

Figure 4A shows the relative light scattering intensities for all the Lo and dLo lipid mixtures studied. As for the undoped PC membrane (Figure 3), the one with 5 mol% PC is almost completely insoluble. For those with 10 and 20 mol% PC, the membranes are incompletely solubilized and the RIFs become less important as the PC content increases. For the two insoluble samples (0 and 5 mol% PC), the DLS experiments show a slight increase in the mean size of the vesicles and a slightly broader monomodal size distribution with increasing TX-100 content (Figure 4B). For the two samples with 10 and 20 mol% PC, the size distribution becomes multimodal at high amounts of TX-100 and the polydispersity index reaches high values, confirming the partial solubilization detected by static light scattering. For all the lipid samples studied here, there is a decrease in the Laurdan GP along the TX-100 titration (Figure A3), but its variation is all the more important as the PC content increases (Table 2, ΔGP). This is consistent with the observed partial solubilization of the membranes containing the highest amounts of PC. Interestingly, while the variation in the GP along the TX-100 titration is the smallest for the Lo 1 sample (Table 2, ΔGP), the perturbation of the membrane is relatively larger (Figure 4C). This relative perturbation logically decreases with increasing PC content due to better detergent insertion in a less packed bilayer. Finally, the residual insoluble fractions seem to correlate with the GP values measured without TX-100 (Figure 4D). The more ordered the Lo phase membrane is in the absence of detergent, the less likely it is to be solubilized.

## 4. Discussion

In the present work, membrane solubilization induced by TX-100 was investigated. Different membrane compositions and phase states were studied along the detergent titration. The expected solubilization profiles were obtained: (i) a classical three-stage model with complete and incomplete solubilization for PC membrane in the Ld phase (Figure 1) and for raft-like mixtures whose membranes exhibit Lo/Ld phase coexistence (Figure 2B), respectively, and (ii) almost complete insolubility for an Lo phase membrane composed of SM and Chol in an equimolar ratio (Figure 3). These solubilization profiles are consistent with those already published [15,22,25,27], but the fluorescence spectroscopy experiments provide new information. Indeed, the C${}_{12}$NBD-PC fluorescence signal indicates that the Lo/Ld phase coexistence is barely unaffected at sub-solubilizing detergent concentrations (Figure 2A) and highlight the vesicle-to-micelle transition (Figure 1 and Figure 2A). Furthermore, the location of the NBD group in the bilayer—linked with its fluorescence intensity—allows to emphasize a detergent–membrane interaction in the case of the insoluble Lo phase membrane (Figure 3). This has already been observed in a similar insoluble SM/Chol membrane [53], but it is highlighted here in a much simpler way. The experiments have also shown that the GP values decrease regardless of the solubilization profiles (Figure A3). This decrease is due to the loosening of membrane packing caused by detergent incorporation, but also to the vesicle-to-micelle transition when it occurs [44,56].

In GPMVs, the ordered phase has been shown to be less ordered than the Lo domains in model membranes, while the disordered phase of the cell-derived membranes is more packed than the Ld phase of model membranes [36,37]. This calls into question the relevance of the pure lipid model systems (GUVs and LUVs) used to study membrane solubilization. Surprisingly, there are very few studies on the solubilization of GPMVs, although this system seems to be a more physiologically relevant system than the model systems mentioned above [33]. This is likely due to the lack of precise control over the composition of GPMVs, although their properties have been shown to be tuned by isolation conditions [57]. Therefore, we used LUVs to study the effect of molecular packing on the extent of solubilization of less ordered Lo phase membranes. To reduce the order of the Lo phase—and to have an order more similar to that found in plasma membranes of living cells—different amounts of PC were added to lipid mixtures composed of SM and Chol in an equimolar ratio (Table 2). PC was chosen because saturated PC species are preferentially found in DRMs [58]. It was found that the moderately PC-doped Lo phase (5 mol% PC) is not solubilized (Figure 4A), although a detergent–membrane interaction is observed, resulting in a decrease in membrane order (Figure 4C and Figure A3). In this case, the DLS experiments show a slight increase in the mean size of the vesicles and a slightly broader monomodal size distribution with increasing TX-100 content (Figure 4B). This behavior corresponds to that of the reference Lo phase. On the contrary, the more disordered Lo phase membranes studied (10 and 20 mol% PC) are partially solubilized (Figure 4A), leading to insoluble fractions with a high polydispersity. The size distribution becomes multimodal and the polydispersity index reaches high values, confirming the partial solubilization detected by static light scattering (Figure 4B). Overall, it is observed that the more ordered the Lo phase membrane is in the absence of detergent, the less likely it is to be solubilized (Figure 4D).

**Figure 5:**
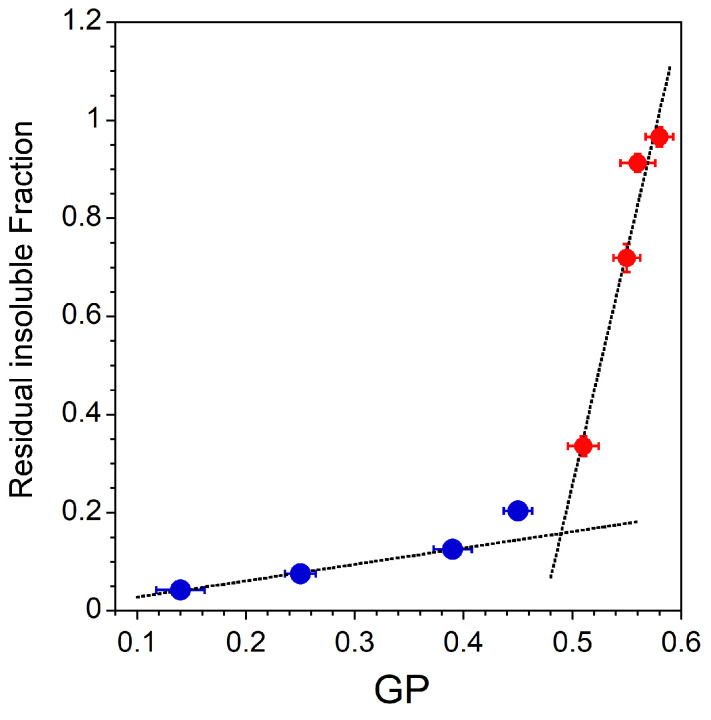
Residual insoluble fractions corresponding to light scattering intensities measured at the highest detergent to lipid ratio (•: Lo-Ld mixtures, Figure 2B; •: Lo and dLo mixtures, Figure 4A) vs. GP values measured without TX-100 (Table 1 and Table 2, $GP_{ini}$). The RIF error bars are shown for values that have been measured three times and the GP error bars are shown for values measured in two sets of five measurements. All measurements were performed at a lipid concentration of 0.1 mM and were recorded at 20 °C.

To summarize the main results, the residual insoluble fraction of the different lipid mixtures is plotted as a function of the GP value measured without TX-100 (Figure 5). As explained before, the RIFs are nothing more than the light scattering intensities measured at the highest detergent to lipid ratio (Figure 2B for Lo-Ld lipid mixtures and Figure 4A for Lo and dLo mixtures). It can be seen in Figure 5 that the higher the GP value, the higher the residual insoluble fraction. These results suggest that the degree of acyl chain packing is a determinant membrane factor for resistance to the action of TX-100. However, the correlation between the initial membrane order ($GP_{ini}$, Table 1 and Table 2) and the extent of solubilization is different depending on the phase state of the membrane. For membranes exhibiting an Lo/Ld phase coexistence, the residual insoluble fractions are weakly dependent on the initial membrane order (Figure 5, •). On the contrary, a very small decrease in the GP value of a membrane in the Lo phase radically modifies the extent of solubilization (Figure 5, •). This high sensitivity of the extent of solubilization to the Lo phase membrane order is important precisely because the ordered phase of GPMVs has been shown to be less ordered than the Lo domains in model systems, and therefore more susceptible to partial solubilization. It has already been recognized that DRMs should not be equated with rafts and cannot provide conclusive evidence of the raft composition or properties. Our results support the assertion that DRMs do not represent living Lo phases.

## Figures and Tables

**Table 1:** Lo-Ld LUV compositions and data.

Sample Name	PC/SM/Chol (mol%) ^(a)^	$\Phi_{L_{o}}$ ^(b)^	${\mathrm{GP}}_{\mathrm{ini}}$ ^(c)^	$T_{\mathrm{misc}}$ ( °C) ^(d)^	RIF ^(e)^	${\mathrm{GP}}_{\mathrm{fin}}$ ^(c)^
Lo-Ld 1	2:1:10	+	0.14	18.5 ±1.2 ^(f)^	+	−0.19
Lo-Ld 2	2:1:20	++	0.25	25.5 ±0.6	+	−0.17
Lo-Ld 3	2:1:35	+++	0.39	33.7 ±0.4	++	−0.20
Lo-Ld 4	1:1:1	No data	0.45	37.4 ±0.2	++	−0.19

^(a)^ With 2 mol% of C${}_{12}$NBD-PC in the membrane when required. ^(b)^ Qualitative Lo phase membrane fraction deduced from GUV images (Figure A1). ^(c)^ GP values without TX-100 ($GP_{ini}$) and with 1.712 mM TX-100 ($GP_{fin}$). Error in GP values: ±0.02. ^(d)^$T_{misc}$ values obtained from a sigmoid fit of the data shown in Figure A2. For $T<T_{misc}$, the membrane exhibits Lo/Ld phase coexistence. ^(e)^ Residual insoluble fraction (RIF): qualitative estimate determined from light scattering intensities measured at the highest detergent to lipid ratio (Figure 2B). ^(f)^ Lo-Ld 1: $T_{misc}<$ 20 °C (see Appendix A.2 for inherent limitations of such curve fitting). GUV fluorescence microscopy experiments (Figure A1, Image B2) and a three-component log-normal decomposition analysis [45] reveal that this membrane exhibits an Lo/Ld phase coexistence at 20 °C.

**Table 2:** Lo and PC-doped Lo LUV compositions and data.

Sample Name	PC/SM/Chol (mol%) ^(a)^	${\mathrm{GP}}_{\mathrm{ini}}$ ^(b)^	$I_{\mathrm{ini}}$ (a.u.) ^(c)^	$T_{\mathrm{misc}}$ ( °C) ^(d)^	RIF ^(e)^	ΔGP ^(f)^
Lo 1	0:1:1	0.57	76.2	60.3 ±3.4	++++	0.50
dLo 1	5:1:1	0.56	103.3	52.9 ±0.9	++++	0.51
dLo 2	10:1:1	0.55	122.1	49.2 ±0.9	+++	0.56
dLo 3	20:1:1	0.51	243.8	44.9 ±0.4	++	0.61

^(a)^ With 2 mol% of C${}_{12}$NBD-PC in the membrane when required. ^(b)^ GP values without TX-100 ($GP_{ini}$). Error in GP values: ±0.02. ^(c)^$I_{ini}$: C${}_{12}$NBD-PC fluorescence intensity maximum without TX-100. ^(d)^$T_{misc}$ values obtained from a sigmoid fit of the data presented in Figure A2. In the temperature range studied, for $T<T_{misc}$, the membrane is in the Lo phase. ^(e)^ Residual insoluble fraction (RIF): qualitative estimate determined from the light scattering intensities measured at the highest detergent to lipid ratio (Figure 4A). ^(f)^$\Delta GP=GP_{ini}-GP_{fin}$.

## Data Availability

Not applicable.

## Abbreviations

The following abbreviations are used in this manuscript:

Chol	Cholesterol
DRM	Detergent-resistant membrane
TX-100	Triton X-100
Lo	Liquid-ordered
Ld	Liquid-disordered
GUV	Giant unilamellar vesicle
SM	Egg yolk sphingomyelin
LUV	Large unilamellar vesicle
GP	Generalized polarization
GPMV	Giant plasma membrane vesicle
PC	Egg yolk L-*α*-phosphatidylcholine
PdI	Polydispersity index
SLS	Static light scattering
RIF	Residual insoluble fraction
DLS	Dynamic light scattering

## Appendix A

### Appendix A.1

Figure A1 shows the compositional phase diagram of the studied three-component bilayer mixtures (A), images of GUVs illustrating the effect of the cholesterol content on the Lo phase fraction $\Phi_{L_{o}}$ in a membrane at 20 °C (B), and images of GUVs with a SM/Chol 50:50 (mol/mol) composition at two temperatures (14 and 60 °C).

### Appendix A.2

The evolution of the GP vs. temperature (15–60 °C) for different lipid compositions is shown in Figure A2. As expected, the GP values decrease with increasing temperature for all lipid compositions tested. LUVs that remain in a single phase over the entire temperature range yield GP vs. *T* curves with a single concavity [45,59]. On the other hand, the mixtures for which a phase transition occurs in the studied temperature range yield a change in concavity with an inflection point corresponding to the miscibility transition temperature ($T_{misc}$) [45,59]. These temperatures were determined by fitting the GP versus temperature curve to the Boltzmann sigmoid function. These temperatures represent an approximate miscibility temperature due to the inherent limitations of such curve fitting.

### Appendix A.3

The effect of the addition of TX-100 on the order of the LUV membrane was monitored by measuring the Laurdan GP. The GP parameter has previously been used to follow the solubilization of liposomes by detergents [56,60]. GP values versus TX-100 concentration are shown in Figure A3 for the different lipid mixtures studied.
